# Characterisation of a mobilisable plasmid conferring florfenicol and chloramphenicol resistance in *Actinobacillus pleuropneumoniae*

**DOI:** 10.1016/j.vetmic.2015.05.020

**Published:** 2015-08-05

**Authors:** Janine T Bossé, Yanwen Li, Tom G Atherton, Stephanie Walker, Susanna M Williamson, Jon Rogers, Roy R Chaudhuri, Lucy A Weinert, Matthew TG Holden, Duncan J Maskell, Alexander W Tucker, Brendan W Wren, Andrew N Rycroft, Paul R Langford

**Affiliations:** a Section of Paediatrics, Department of Medicine, Imperial College London, St. Mary’s Campus, London, W2 1PG, UK; b Animal and Plant Health Agency (APHA) Bury St Edmunds, Rougham Hill, Bury St Edmunds, Suffolk, IP33 2RX, UK; c Department of Veterinary Medicine, University of Cambridge, Madingley Road, Cambridge, CB3 0ES, UK; d The Wellcome Trust Sanger Institute, Wellcome Trust Genome Campus, Hinxton, Cambridge CB10 1SA, UK; e Faculty of Infectious & Tropical Diseases, London School of Hygiene & Tropical Medicine, Keppel Street, London, WC1E 7HT, UK; f Department of Pathology and Pathogen Biology, The Royal Veterinary College, Hawkshead Campus, Hatfield, Hertfordshire, AL9 7TA, UK

## Abstract

The complete nucleotide sequence of a 7.7 kb mobilisable plasmid (pM3446F), isolated from a florfenicol resistant isolate of *Actinobacillus pleuropneumoniae*, showed extended similarity to plasmids found in other members of the *Pasteurellaceae* containing the *floR* gene as well as replication and mobilisation genes. Mobilisation into other *Pasteurellaceae* species confirmed that this plasmid can be transferred horizontally.

## Highlights

• First complete sequence of a *floR* plasmid from *Actinobacillus pleuropneumoniae*
• Extended similarity to *floR* plasmids in other *Pasteurellaceae* species
• Conjugal transfer between between species confirmed

## 1 Introduction

*Actinobacillus pleuropneumoniae*, one of the main pathogens contributing to swine respiratory disease throughout the world, has been used as an indicator organism in surveillance studies of antimicrobial resistance in bacteria of animal origin (de Jong et al., 2014; Hendriksen et al., 2008). Tetracyclines, beta-lactams, and trimethoprim/sulphonamides are the commonest frontline treatments for porcine pleuropnuemonia in Europe (European Medicines Agency, 2012), however, levels of resistance are increasing (de Jong et al., 2014; Vanni et al., 2012). To a lesser extent, florfenicol, a fluorinated thiamphenicol derivative licensed to treat respiratory diseases of pigs and cattle in Europe since 2000 and 1995, respectively (Kehrenberg et al., 2004), has been used. Most surveys of *A. pleuropneumoniae* antimicrobial susceptibility have shown that nearly all isolates are susceptible to florfenicol, except in Korea where resistance levels of 34% were recently reported (Gutiérrez-Martín et al., 2006; Kucerova et al., 2011; Priebe and Schwarz, 2003; Shin et al., 2005; Vanni et al., 2012; Yoo et al., 2014).

A chloramphenicol/florfenicol efflux pump, encoded by *floR* (Schwarz et al., 2004), has been detected by PCR in florfenicol-resistant *A. pleuropneumoniae* isolates (Kucerova et al., 2011; Yoo et al., 2014), and this resistance was transferrable to *Escherichia coli* (Yoo et al., 2014), however no plasmid was characterised.

Here we describe the isolation and characterisation of a 7.7 kb florfenicol resistance plasmid from a clinical isolate of *A. pleuropneumoniae*. Comparative sequence analysis of this plasmid with others reported as mediating florfenicol resistance in bovine isolates of *Pasteurella multocida* (Kehrenberg and Schwarz, 2005; Kehrenberg et al., 2008) and *Mannheimia haemolytica* (Katsuda et al., 2012) is also described.

### 1.1 Material and methods

#### 1.1.1 Bacterial isolates and MIC measurements

*A. pleuropneumoniae* MIDG3446, a serovar 2 clinical isolate, was originally cultured from pneumonic lungs of a pig in Greece in 2010 and archived at the then Animal Health and Veterinary Laboratory Agency (now Animal and Plant Health Agency) in England. Minimum inhibitory concentrations (MIC) for florfenicol and chloramphenicol were determined by agar dilution using Chocolate Mueller-Hinton plates, with *A. pleuropneumoniae* ATCC 27090, *Histophilus somni* ATCC 70025 and *Staphylococcus aureus* ATCC 29213 used as quality control strains (CLSI, 2008).

#### 1.1.2 Plasmid isolation and confirmation of floR by PCR

Plasmid DNA was isolated using a Qiaprep Spin Miniprep kit (Qiagen) according to the manufacturer's protocol. The presence of the *floR* gene was confirmed by PCR using primers floR_for (CGACGCCCGCTATGATCCAACTC) and floR_rev (CCCAAAAAGCCGGACTCGCGAAG). Initially, florfenicol resistance was transferred to *E. coli* Stellar cells (Clontech) by heat shock with the MIDG3446 plasmid extract, according to the manufacturer's protocol, with selection of transformants on LB agar supplemented with 10 μg/ml florfenicol. Subsequently, mobilisation into selected *Pasteurellaceae* strains was assessed using a mating protocol previously described (Bossé et al., 2009; Bossé et al., 2015). Recipient strains included nalidixic acid resistant derivatives of *M. haemolytica* (MIDG1579Nal^R^), *P. multocida* (MIDG1570 Nal^R^), and *Haemophilus parasuis* (MIDG3176Nal^R^), as well as the NAD-independent *A. pleuropneumoniae* isolate, MIDG2331Δ*ureC*::*nadV*, previously described (Bossé et al., 2009; Bossé et al., 2014). Transconjugants were selected on Brain Heart Infusion agar (with or without 0.01% NAD or 20 μg/ml nalidixic acid, as appropriate) supplemented with 2 μg/ml florfenicol. Transformants and transconjugants were tested by PCR for the presence of *floR*, as above, and for the *nadV* gene, as previously described (Bossé et al., 2015), where appropriate. MICs for florfenicol and chloramphenicol were determined, as above, for recipient strains +/− plasmid.

### 1.2 Plasmid sequence

A plasmid carrying the *floR* gene was isolated from a selected *E. coli* transformant (above) and the complete nucleotide sequence was determined using a primer walking strategy. Sequence analysis was carried out using BLASTn and BLASTx. Alignments with other *floR* plasmids were done using ClustalW. The sequence of the *A. pleuropneumoniae floR* plasmid (pM3446F) has been deposited to Genbank (accession number KP696484).

## 2 Results and discussion

**Fig. 1:**
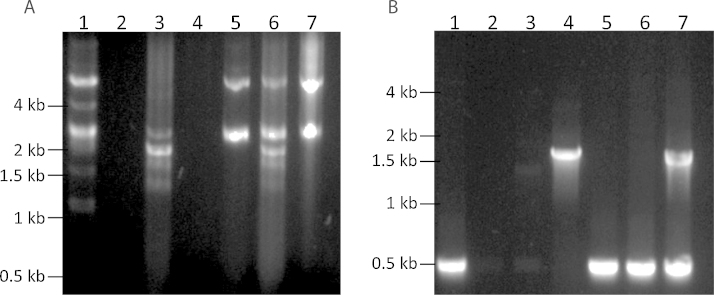
Transfer of florfenicol resistance plasmid from *A. pleuropneumoniae* MIDG3446 by transformation into *E. coli* Stellar, or mating into *M. heamolytica* MIDG1579 and *A. pleuropneumoniae* MIDG2331 Δ*ureC*::*nadV*. (A) Comparison of plasmid extracts from donor strain MIDG3446 (lane 1), recipient strains *E. coli* Stellar, *M. heamolytica* MIDG1579 and *A. pleuropneumoniae* MIDG2331 Δ*ureC*::*nadV* (lanes 2-4), and respective florfenicol resistant transformant/transconjugants showing transfer of plasmid pM3446F into *E. coli* Stellar, *M. heamolytica* MIDG1579, and *A. pleuropneumoniae* MIDG2331 Δ*ureC*::*nadV* (lanes 5-7) (B) Multiplex PCR amplification of *floR* (510-bp amplicon) and *nadV* (1.5 kb amplicon) from *A. pleuropneumoniae* MIDG3446 (lane 1), *E. coli* Stellar (lane 2), *M. heamolytica* MIDG1579 (lane 3), *A. pleuropneumoniae* MIDG2331 Δ*ureC*::*nadV* (lane 4), *E. coli* Stellar + pM3446F (lane 5), *M. heamolytica* MIDG1579 + pM3446F (lane 6), *A. pleuropneumoniae* MIDG2331 Δ*ureC*::*nadV* + pM3446F (lane 7).

A serovar 2 isolate of *A. pleuropneumoniae*, MIDG3446, was found to be resistant to florfenicol and chloramphenicol (Table 1). Analysis of DNA from MIDG3446 revealed multiple small plasmids, one of which when transformed into *E. coli* Stellar cells (Clontech), conferred resistance to florfenicol and chloramphenicol (Fig. 1; Table 1). Additionally, mobilisation from MIDG3446 was successful into *A. pleuropneumoniae* MIDG2331Δ*ureC*::*nadV* and *M. haemolytica* MIDG1579Nal^R^, but not *P. multocida* MIDG1570Nal^R^ nor *H. parasuis* MIDG3176Nal^R^. The location of the genes encoding the conjugation machinery was not determined, and no attempts were made to optimise mating conditions, nor were other isolates of these species tested. PCR confirmed the presence of *floR* in transformants and transconjugants (Fig. 1), and MICs for florfenicol and chloramphenicol, determined as above, for recipient strains +/− plasmid indicated transfer of resistance with the plasmid (Table 1).

**Fig. 2:**
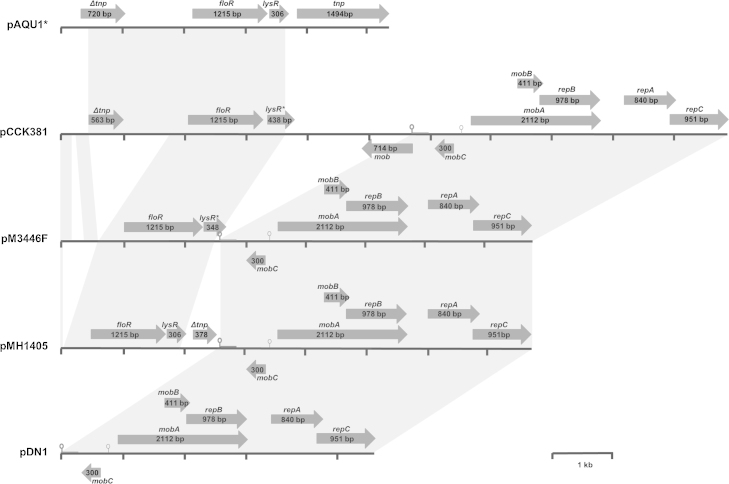
Schematic comparison of florfenicol resistance plasmid pM3446F from *A. pleuropneumoniae* with plasmids pAQU1 (from *Photobacterium damselae*, subsp. *damselae*), pCCK381 and pMH1405 (from related *Pasteurellaceae*), and pDN1 (from *Dichelobacter nodosus*). Note: only 5.3 kb (from bases 138014–143017 of 204052 total) of pAQU1 is shown. Reading frames are indicated by arrows, with arrowheads showing direction of transcription (*floR*: florfenicol resistance; *lysR*: transcriptional regulator; lysR*: partial *lysR*; *tnp*: transposase; Δ*tnp*: truncated transposase; *mob*, *mobA*, *mobB*, *mobC*: plasmid mobilisation; *repA*, *repB*, *repC*: plasmid replication). The predicted origins of replication (*oriV*; ) and transfer (*oriT*; ) are shown. Grey blocks between sequences indicate ≥ 98% nucleotide sequence identity. A distance scale in kb is shown.

The complete 7,709-bp sequence of pM3446F, isolated from a selected *E. coli* transformant, was determined using a primer walking strategy. Sequence analysis using BLASTn and BLASTx (http://blast.ncbi.nlm.nih.gov/Blast.cgi) revealed extended similarity to pMh1405 (AB621552), a 7,674-bp plasmid isolated in 2009 from an *M. haemolytica* strain recovered from a calf in Japan (Katsuda et al., 2012), and to a lesser extent to pCCK381 (AJ871969), a 10,874-bp plasmid isolated in 2005 from a *P. multocida* strain recovered from a calf in Thirsk, England (Kehrenberg and Schwarz, 2005), and subsequently from bovine and porcine isolates of *P. multocida* in Germany (Kehrenberg et al., 2008). All three *Pasteurellaceae*-derived plasmids (Fig. 2) have virtually identical replication (*repA-C*) and mobilisation genes (*mobA-C*), as well as *oriV* and *oriT* sequences, as those comprising pDN1 (Fig. 2), a 5,112-bp RSF1010-like IncQ broad-host-range plasmid (Y19120) isolated from *Dichelobacter nodosus*, the causative agent of ovine footrot (Kehrenberg and Schwarz, 2005; Rawlings and Tietze, 2001; Whittle et al., 2000). However, whereas pDN1 does not encode resistance genes, these *Pasteurellaceae* plasmids contain differing fragments of what was initially described as Tn*floR*, a transposable element encoding *floR*, a *lysR* transcriptional regulator, and the *tnpA* transposase (Doublet et al., 2005), and subsequently shown to be an IS*CR2*-*floR* element (Toleman et al., 2006). Similar sequences have been found in various plasmids and chromosomes of different bacterial species, suggesting recombination and/or deletion events following integration have given rise to different truncated forms of the genes flanking *floR* (Doublet et al., 2005; Schwarz et al., 2004; Toleman et al., 2006).

The sequences flanking *floR* in these *Pasteurellaceae* plasmids show greatest similarity to those found in pAQU1 (AB571865), a 200 kb conjugative plasmid isolated from *Photobacterium damselae*, subsp. *damselae* in Japan (Nonaka et al., 2012). In Figure 2, only the sequence around the IS*CR2*-*floR* element of pAQU1 is shown (from bases 138014 to 143017 in the Genbank annotated sequence), with the Δ*tnp* gene upstream of *floR* almost identical to the last 720 bases of the *tnp* gene downstream. In pCCK381, in addition to the partial IS*CR2*-*floR* (99% identity with pAQU1 bases 138160–141381), the 2062 bases comprising the end of the *lysR* gene to the start of the *oriV* are 99% identical to bases 146509–148591 of pAQU1. Whereas, upstream of the Δ*tnp* gene in pCCK381, there are sequences 99-100% identity with other regions (bases 148616–148778 and bases 148992–149190) of pAQU1. In pMh1405, the 2.5 kb upstream of *oriV* shares 99% identity with bases 139426–141904 of pAQU1 (a different deletion fragment of IS*CR2*-*floR* than that seen in pCCK381); and in pM3446F no *tnp* sequences remain (with bases 622–2522 of pM3446F being 99% identical to bases 139426–141904 of pAQU1). An orf downstream of *floR* in pM3446F shows partial identity with *lysR*, with the first 223/348 bp being 100% identical to bases 141107–141329, and bases 214–334 of the orf having 98% identity with bases 148514–148633, of pAQU1. In addition, bases 25– 597 of pM3446F are 99% identical to bases 148616–149190 of pAQU1, parts of which are conserved in the sequence upstream of the Δ*tnp* gene in pCCK381. These data suggest that these *Pasteurellaceae* plasmids share a common origin, with sequences from pAQU1 (or a related plasmid) having integrated into pDN1, and subsequent deletions/rearrangements of the inserted sequences.

Two further *Pasteurellaceae floR* plasmids have been identified: pCCK1900 (NC_011378), a 10,226-bp plasmid from a porcine isolate of *P. multocida* in Germany (Kehrenberg et al., 2008) that in encodes resistance to florfenicol (*floR*), sulphonamide (*sul2*), and streptomycin (*strA*, *strB*); pCCK13698 (NC_007800), a 14,969-bp plasmid from a bovine isolate of *Bibersteinia trehalosi* in France (Kehrenberg et al., 2006) that encodes resistance to florfenicol (*floR*), chloramphenicol (*catA3*) and sulphonamide (*sul2*). The sequence of pCCK1900 shows simple integration of the *floR* and *lysR* genes into an RSF1010 backbone (Kehrenberg et al., 2008). Although pCCK13698 shares some *rep* and *mob* gene sequences, as well as the *sul2* gene, with RSF1010, it shows more extensive rearrangements with sequences from other plasmids including pHS-rec from *H. parasuis* (Lancashire et al., 2005) and pMVSCS1 from *Mannheimia varigena* (Kehrenberg and Schwarz, 2002), and the *floR* and *lysR* genes from IS*CR2*-*floR* (Kehrenberg et al., 2006). Both pCCK1900 and pCCK13698 appear to have arisen separately from the pDN1-like plasmids described above.

## 3 Conclusion

In summary, to our knowledge, this is the first report of a complete sequence of a mobilisable florfenicol resistance plasmid from *A. pleuropneumoniae*. Structural analysis of pM3446F revealed extensive similarity to two florfenicol resistance plasmids found in other members of the *Pasteurellaceae*, and mating experiments confirmed the ability to mobilise between species. This highlights the importance of continued surveillance of florfenicol susceptibility in *A. pleuropneumoniae* and other Gram-negative pathogens that may co-exist within the respiratory tract of pigs.

### 3.1 Funding

This work was supported by a Longer and Larger (LoLa) grant from the Biotechnology and Biological Sciences Research Council (grant numbers BB/G020744/1, BB/G019177/1, BB/G019274/1 and BB/G018553/1), the UK Department for Environment, Food and Rural Affairs, and Zoetis (formerly Pfizer Animal Health) awarded to the Bacterial Respiratory Diseases of Pigs-1 Technology (BRaDP1T) consortium. MTGH was supported by Wellcome Trust grant 98051. The MIC work was funded from the former AHVLA's Research and Development Internal Investment Fund RD0030c . The funders had no role in study design, data collection and analysis, decision to publish, or preparation of the manuscript.

## Figures and Tables

**Table 1:** Antimicrobial susceptibility for strains with and without plasmids.

Strain	Species	MIC (μg/ml)		Source or Reference
		Florfenicol	Chloramphenicol	
MIDG3446	Ap^a^	8	8	This study
MIDG2331Δ*ureC*::*nadV*	Ap	2	2	(Bossé et al., 2014)
MIDG2331Δ*ureC*::*nadV* + pM3446F	Ap	8	8	This study
MIDG1579	Mh^b^	1	1	(Bossé et al., 2009)
MIDG1579 + pM3446F	Mh	16	16	This study
Stellar	Ec^c^	2	4	Clontech
Stellar + pM3446F	Ec	16	32	This study

a Ap =* A. pleuropneumoniae*.

b Mh = *M. haemolytica*.

c Ec = *E. coli.*
