# Corrigendum to ‘Characterisation of a mobilisable plasmid conferring florfenicol and chloramphenicol resistance in Actinobacillus pleuropneumoniae’ [Veterinary Microbiology 178 (2015) 279–282]

**DOI:** 10.1016/j.vetmic.2015.07.002

**Published:** 2015-09-30

**Authors:** Janine T. Bossé, Yanwen Li, Tom G. Atherton, Stephanie Walker, Susanna M. Williamson, Jon Rogers, Roy R. Chaudhuri, Lucy A. Weinert, Matthew T.G. Holden, Duncan J. Maskell, Alexander W. Tucker, Brendan W. Wren, Andrew N. Rycroft, Paul R. Langford, BRaDP1T consortium

**Affiliations:** a Section of Paediatrics, Department of Medicine, Imperial College London, St. Mary’s Campus, London W2 1PG, UK; b Animal and Plant Health Agency (APHA) Bury St Edmunds, Rougham Hill, Bury St Edmunds, Suffolk IP33 2RX, UK; c Department of Veterinary Medicine, University of Cambridge, Madingley Road, Cambridge CB3 0ES, UK; d The Wellcome Trust Sanger Institute, Wellcome Trust Genome Campus, Hinxton, Cambridge CB10 1SA, UK; e Faculty of Infectious & Tropical Diseases, London School of Hygiene & Tropical Medicine, Keppel Street, London WC1E 7HT, UK; f Department of Pathology and Pathogen Biology, The Royal Veterinary College, Hawkshead Campus, Hatfield, Hertfordshire AL9 7TA, UK

The authors regret that the BraDP1T consortium were not named in the author listing of their original paper. The correct listing appears above.

Please see below for a full list of Collaborators (16):

Maskell DJ, Tucker AW, Peters SE, Weinert LA, Wang JT, Luan SL, Chaudhuri RR, Rycroft AN, Maglennon GA, Matthews D, Langford PR, Bossé JT, Li Y, Wren BW, Cuccui J, Terra VS.

The authors would like to apologise for any inconvenience caused.

